# Mechanistic Insight into the Regulation of Lipoxygenase-Driven Lipid Peroxidation Events in Human Spermatozoa and Their Impact on Male Fertility

**DOI:** 10.3390/antiox10010043

**Published:** 2020-12-31

**Authors:** Jessica L. H. Walters, Amanda L. Anderson, Sarah J. Martins da Silva, R. John Aitken, Geoffry N. De Iuliis, Jessie M. Sutherland, Brett Nixon, Elizabeth G. Bromfield

**Affiliations:** 1 Priority Research Centre for Reproductive Science, Schools of Biomedical Science & Pharmacy and Environmental & Life Sciences, The University of Newcastle, Callaghan, NSW 2308, Australia; jwalters1@uon.edu.au (J.L.H.W.); amanda.anderson@newcastle.edu.au (A.L.A.); john.aitken@newcastle.edu.au (R.J.A.); geoffry.deiuliis@newcastle.edu.au (G.N.D.I.); jessie.sutherland@newcastle.edu.au (J.M.S.); brett.nixon@newcastle.edu.au (B.N.); 2 Hunter Medical Research Institute, Pregnancy and Reproduction Program, New Lambton Heights, NSW 2305, Australia; 3 Reproductive Medicine Research Group, School of Medicine, University of Dundee, Dundee DD1 9SY, UK; s.martinsdasilva@dundee.ac.uk; 4 Assisted Conception Unit, Ninewells Hospital and Medical School, Dundee DD1 9SY, UK; 5 Discipline of Biochemistry and Cell Biology, Faculty of Veterinary Medicine, Utrecht University, 3584 CM Utrecht, The Netherlands

**Keywords:** male infertility, lipid peroxidation, spermatozoa, lipoxygenase

## Abstract

A prevalent cause of sperm dysfunction in male infertility patients is the overproduction of reactive oxygen species, an attendant increase in lipid peroxidation and the production of cytotoxic reactive carbonyl species such as 4-hydroxynonenal. Our previous studies have implicated arachidonate 15-lipoxygenase (ALOX15) in the production of 4-hydroxynonenal in developing germ cells. Here, we have aimed to develop a further mechanistic understanding of the lipoxygenase-lipid peroxidation pathway in human spermatozoa. Through pharmacological inhibition studies, we identified a protective role for phospholipase enzymes in the liberation of peroxidised polyunsaturated fatty acids from the human sperm membrane. Our results also revealed that arachidonic acid, linoleic acid and docosahexanoic acid are key polyunsaturated fatty acid substrates for ALOX15. Upon examination of ALOX15 in the spermatozoa of infertile patients compared to their normozoospermic counterparts, we observed significantly elevated levels of ALOX15 protein abundance in the infertile population and an increase in 4-hydroxynonenal adducts. Collectively, these data confirm the involvement of ALOX15 in the oxidative stress cascade of human spermatozoa and support the notion that increased ALOX15 abundance in sperm cells may accentuate membrane lipid peroxidation and cellular dysfunction, ultimately contributing to male infertility.

## 1. Introduction

The molecular understanding of male infertility has emerged as a crucial area of research in view of the growing burden of infertility that afflicts an estimated 15% of couples globally. While there are many underlying causes of male infertility (discussed at length in [1]), strong associations have been identified linking male infertility with increased levels of cellular oxidative stress as measured by reactive oxygen species (ROS) production and resultant signatures of lipid peroxidation within spermatozoa [2,3,4,5,6,7]. Accordingly, in vitro studies have established causality between the induction of cellular oxidative stress and lipid peroxidation, and a range of adverse functional sequelae, including deficits in sperm motility, capacitation, acrosome reaction rates and interactions with the zona pellucida [7,8,9]. Regrettably, however, the knowledge that oxidative stress and ROS overproduction is intimately tied to sperm dysfunction has done little to provide improved treatment outcomes to male infertility patients. Indeed, despite the completion of numerous clinical trials designed to assess the therapeutic utility of antioxidants, these interventions have thus far failed to deliver on their expectations [10,11,12]. Whilst the reasons for these disappointing outcomes are undoubtedly complex, factors such as a failure to target appropriate patient cohorts, the use of antioxidant formulations that are not tailored toward the specific stress experienced in the male germline, and inconsistencies in measured outcomes all contribute [13]. Irrespective, the limitations of our current armoury of antioxidant strategies suggest that more targeted approaches, which exploit our mechanistic knowledge of male germ cell biology, are urgently needed.

Although lipid peroxidation levels are commonly reported as elevated in dysfunctional spermatozoa following an in vitro induction of oxidative stress [6] or in infertile patients [14,15], the key mechanisms responsible for driving the lipid peroxidation cascade downstream of ROS production, remain poorly characterised in human spermatozoa. The literature pertaining to the lipid peroxidation pathway in the male germ line typically draw on analogies with somatic cell behaviour, thus leaving many of the fundamental aspects of this pathway uncharacterised in mature human spermatozoa. This remains an important avenue for research since the structure and function of the male gamete fundamentally differs from that of their somatic cell counterparts. Such differences extend to the unique highly specialised architecture of the spermatozoon in which the cytoplasmic content, and hence antioxidant capacity, is severely limited combined with the fact that these cells are transcriptionally and translationally silent and thus incapable of repairing cellular damage should it arise [16,17]. Accordingly, a more thorough examination of the molecular mechanisms that underpin the initiation and propagation of lipid peroxidation within human sperm cells is warranted.

In seeking to address this unmet need, our previous research has focused on the characterisation of the lipoxygenase enzyme, arachidonate 15-lipoxygenase (ALOX15), that is responsible for catalysing lipid peroxidation via the oxygenation of polyunsaturated fatty acid (PUFA) substrates [18,19,20]. Ultimately, ALOX15 facilitates the production of cytotoxic lipid aldehydes, causing cellular damage that can perpetuate further oxidative insults into widespread collateral damage throughout the germ cell [13]. In our studies, we have confirmed that ALOX15 holds a central role in the lipid peroxidation cascade within mature human spermatozoa. Indeed, selective ALOX15 inhibition via PD146176 treatment, attenuates both cellular oxidative stress and lipid peroxidation and maintains sperm functionality [8]. In view of these promising data, this study was designed to generate additional mechanistic insights into the regulation of lipoxygenase driven lipid peroxidation cascades in human spermatozoa and examine the relative abundance of ALOX15 in the spermatozoa of infertile men. Specifically, we sought to examine the profile of PUFA substrates metabolised by ALOX15, as well as the involvement of phospholipase enzymes in the upstream liberation of these PUFAs from within the damaged sperm plasma membrane. With the goal of formulating effective interventions to prevent the demise of sperm cells in the face of an oxidative insult, we also evaluated whether ALOX15 works in tandem with additional members of the lipoxygenase family in mediating human sperm lipid peroxidation. Overall, the insights from this study of the lipoxygenase-lipid peroxidation pathway operating in human sperm represent important advances toward the long-term development of tailored therapeutic strategies to reduce the burden of male infertility associated with oxidative stress.

## 2. Materials and Methods

### 2.1. Ethics

All reported studies involving human semen samples were performed in accordance with the University of Newcastle Human Ethics Committee guidelines (Approval No. H-2013-0319). Volunteers consisted of a panel of healthy donors with normozoospermic semen parameters as determined by routine andrological assessment in accordance with WHO criteria. Patient recruitment and consent was in accordance with the Human Fertilisation and Embryology Authority (HFEA) Code of Practice (version 8) and under local ethical approval (13/ES/0091) from East of Scotland Research Ethics Service (EoSRES) REC1. All donors and patients provided informed written consent for the use of their samples.

### 2.2. Reagents

Unless specified below, all chemical reagents were of research grade and were obtained from Merck (Kenilworth, NJ, USA). Those chemicals sourced from Thermo Fisher Scientific (Waltham, MA, USA) included BODIPY 581/591 C11 and the LIVE/DEAD viability reagent. The lipoxygenase inhibitors, PD146176, BW-B-70C and 2-TEDC were purchased from Tocris Bioscience (Avonmouth, Bristol, UK). ML355 and docosapentanoic acid were supplied by Cayman Chemical (Ann Arbor, MI, USA). Anti-ALOX15 antibodies were purchased from Abcam (Cambridge, MA, USA), while GE Healthcare (Chicago, IL, USA) supplied nitrocellulose membranes and Percoll. Tris-HCl was obtained from ICN Biochemicals (Castle Hill, NSW, Australia) and Hams-F10 was bought from MP Biomedicals (Irvine, CA, USA). The catalogue numbers of antibodies and details for the working solutions of each inhibitor and probe are provided in Supplementary Table S1.

### 2.3. Preparation of Human Spermatozoa

Human sperm samples were prepared following a 30 min liquefaction period. To separate high- and low-quality sperm populations, raw semen samples were individually layered onto a 40%/80% discontinuous Percoll gradient and centrifuged at 500× *g* for 30 min as previously described [21]. The pellet formed at the bottom of the tube comprises the sub-population of high-quality spermatozoa within each sample, with the less dense cells partitioning at the interface of the 40% and 80% fractions representing lower-quality cells as defined by poorer semen parameters and reduced functional competence [22,23]. Following isolation of this sperm population, the cells were resuspended in 5 mL of non-capacitating Biggers Whitten and Whittingham (NC BWW) media [24] lacking bicarbonate and centrifuged for a further 15 min (500× *g*). Sperm were then resuspended in NC BWW medium at a concentration of 10 × 10^6^ cells/mL in preparation for experimental analyses. Commercially available media were used to prepare patient samples at Ninewells Assisted Conception Unit, Dundee. Spermatozoa were separated from semen by density gradient centrifugation (40%/80%) using PureCeption diluted with HEPES-buffered human tubal fluid (HTF) (Cooper Surgical; Måløv, Denmark). After centrifugation, the pellet was washed by centrifugation (500× *g*; 10 min) in 4 mL Quinn’s Advantage Medium with HEPES. If the samples were assigned for IVF, the supernatant was discarded following centrifugation and the pellet resuspended in Quinn’s Advantage Fertilization medium. If the sample was allocated for ICSI, the cells were washed and prepared in Quinn’s Advantage Medium with HEPES and HAS.

### 2.4. Induction of Lipid Peroxidation and Inhibitor Treatment

In the majority of the reported studies, the induction of lipid peroxidation was achieved by treating spermatozoa with arachidonic acid (AA). Given this, a dose–response pilot study was performed (5–50 µM) to establish an effective AA concentration with which to upregulate lipid peroxidation without compromising sperm viability. Following these results, we elected to use a treatment regimen of 5 µM AA for 30 min at 37 °C. Broad spectrum phospholipase inhibition was achieved using the phospholipase A_2_ inhibitor, arachidonyl trifluoromethyl ketone (AACOCF_3_) [25,26]. Spermatozoa were pre-treated with AACOCF_3_ (0.05–5 µM) for 30 min at 37 °C prior to the induction of lipid peroxidation via challenge with either H_2_O_2_ (1 mM, 1 h, 37 °C) or arachidonic acid (AA; 5 µM, 30 min, 37 °C). Similarly, lipoxygenase enzyme activity was inhibited with a range of selective and broad-spectrum pharmacological reagents. Specifically, ALOX15 was selectively inhibited with PD146176 (6,11-Dihydro[1]benzothiopyrano[4,3-b]indole), ALOX12 with ML355 (*N*-2-benzothiazolyl-4-[[(2-hydroxy-3-methoxyphenyl)methyl]amino]-benzenesulfonamide), and ALOX5 with BW-B 70C (*N*-[3-[3-(-fluorophenoxy)phenyl]-1-methyl-2-propenyl]-*N*-hydroxyurea). Simultaneous inhibition of ALOX5, ALOX12 and ALOX15 was accomplished with the broad spectrum lipoxygenase inhibitor, 2-TEDC (2-(1-thienyl)ethyl 3,4-dihydroxybenzylidenecyanoacetate). Each of these inhibitors was used at concentrations of between 0.05 and 5 µM and pre-treatment of sperm was completed for 30 min prior to inducing lipid peroxidation with either arachidonic acid (AA; 5 µM, 30 min, 37 °C), linoleic acid (LA; 300 µM, 30 min, 37 °C) or docosahexanoic acid (DHA; 5 µM, 30 min, 37 °C) challenge. Importantly, all such PUFAs are present in human sperm plasma membranes [27] and are documented in a broad range of cell types to be effective inducers of cellular stress [28,29,30,31,32]. Negative controls for lipid peroxidation were also completed using the treatments of caprylic acid (5–5000 µM) and docosapentanoic acid (5–100 µM) (neither of which are recognised lipoxygenase substrates), without the addition of a lipoxygenase inhibitor. In the final 10 min of each treatment, the sperm suspension was supplemented with a LIVE/DEAD viability stain (diluted 1:10,000 in NC BWW) in order to facilitate assessment of sperm cell viability.

### 2.5. Assessment of Lipid Peroxidation

Following isolation of human spermatozoa, cells were incubated with the fluorescent probe BODIPY 581/591 C11 (5 µM) for 30 min at 37 °C as previously described [8]. Based on shifts in spectral emission maxima, this probe differentiates cells with undamaged membranes as opposed to those that have experienced lipid peroxidation. Following incubation with BODIPY 581/591 C11, sperm were washed free of this probe by dilution into NC BWW and centrifugation in triplicate (500× *g*, 3 min). Treatments to induce lipid peroxidation or enzyme inhibition were then completed as previously described. Spermatozoa were then washed in duplicate (500× *g*, 3 min) and analysed by flow cytometry as previously described [6]. A minimum of 10,000 cells per treatment were assessed using a flow cytometer to determine the levels of live BODIPY fluorescent positive cells within each sample. BODIPY positive cells were determined via spectra emission shifts from 590 to 510 nm. Viability was confirmed using the LIVE/DEAD fluorescent marker where positive fluorescence indicated non-viable spermatozoa.

### 2.6. Comparison of ALOX15 Expression in Infertile and Fertile Spermatozoa

Human sperm cells were isolated using Percoll or PureCeption density gradient centrifugation as described above. Aside from those cells dedicated to the analysis of lipid peroxidation levels, the remainder of each sample was prepared for protein extraction. For this purpose, spermatozoa were resuspended in sodium dodecyl sulfate (SDS) extraction buffer (0.375 M Tris pH 6.8, 2% *w*/*v* SDS, 10% *w*/*v* sucrose, protease inhibitor cocktail) and boiled for 5 min at 100 °C as previously described [8,33]. Samples were then centrifuged for 15 min at 17,000× *g*, 4 °C and the solubilised protein recovered in the supernatants was quantified using a DC protein quantification kit as per the manufacturer’s instructions (Bio-Rad Laboratories, Hercules, CA, USA). Protein samples were diluted as appropriate in SDS-PAGE sample buffer (2% *v*/*v* mercaptoethanol, 2% *w*/*v* SDS, and 10% *w*/*v* sucrose in 0.375 M Tris, pH 6.8 with bromophenol blue) and electrophoresed on precast 4–20% Tris glycine gel for 1 h at 150 V (Bio-Rad Laboratories, Hercules, CA, USA). Resolved proteins were then transferred to a nitrocellulose membrane via electroblotting for 1 h at a constant current of 350 mA. Following protein transfer, the nitrocellulose membranes were blocked for 1 h with 5% skim milk powder prepared in Tris buffered saline (TBS) supplemented with 0.1% *v*/*v* polyoxyethylenesorbitan monolaurate (Tween-20) and then incubated overnight (at 4 °C) with either an anti-ALOX15 primary antibody or anti-4HNE primary antibodies (Supplementary Table S1). Following overnight exposure to the primary antibodies, membranes were washed in triplicate (TBST, 10 min) and then labelled with a goat-anti-rabbit horseradish peroxidase (HRP)-conjugated secondary antibody (diluted to 0.13 μg/mL in 1% skim milk/TBST). Following secondary antibody incubation, membranes were again washed in triplicate and developed using an enhanced chemiluminescence detection kit as per the manufacturer’s recommendations (GE Healthcare).

### 2.7. Statistical Analysis

Statistical analyses were completed using JMP statistical software (SAS Institute Inc., Cary, NC, USA). The number of replicates (biological and/or technical) is specified in each figure legend. Importantly, all data sets were initially assessed for normal distribution using a goodness of fit Shapiro–Wilk test. If normality measures were met, parametric statistical testing using unpaired Student’s *t*-tests were completed. In the event that data were non-normally distributed, non-parametric statistical testing was employed using the Wilcoxon test. The results for each of these tests are annotated in the appropriate figures with significance being denoted by the inclusion of asterisks where: * *p* < 0.05, ** *p* < 0.01, and *** *p* < 0.001.

## 3. Results

### 3.1. Arachidonic Acid Treatment Induces Lipid Peroxidation and Cell Death

**Figure 1:**
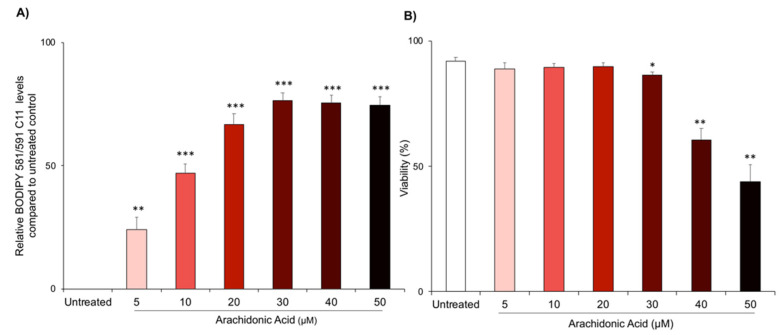
Induction of lipid peroxidation with AA. High quality human sperm cells were obtained via Percoll density centrifugation and then exposed to the BODIPY 581/591 C11 probe (5 µM, 30 min, 37 °C) to assess lipid peroxidation (**A**). Cells were then treated with AA at increasing concentrations (5–50 µM, 30 min, 37 °C). To assess viability (**B**), LIVE/DEAD was added to each of sample in the final 10 min of AA treatment. Sperm cells were then washed of treatment and analysed by flow cytometry (counting a minimum of 10,000 cells). Statistical analysis was completed on *n* = 5 biological replicates, with all data presented as mean + SEM and with statistically significant changes, compared to the untreated control, being denoted by * *p* < 0.05, ** *p* < 0.01 and *** *p* < 0.001.

To pharmacologically manipulate elements of the lipid peroxidation pathway in human sperm cells, lipid peroxidation was first induced by directly exposing sperm cells to arachidonic acid. This treatment was chosen on the basis of the specificity of PUFAs to the lipid peroxidation pathway [34,35] and the documented capacity of AA to induce oxidative stress [30,31,32,36]. To establish the efficacy of this approach, a dose–response was performed using doses of 5–50 µM AA (Figure 1). Significant increases in lipid peroxidation levels (as measured by BODIPY 581/591 C11 flow cytometry) were observed for all AA treatment concentrations (Figure 1A; *p* < 0.01 or *p* < 0.001), while cell death remained stable until AA treatments reached a concentration of 30 µM (Figure 1B). Thereafter, cell viability was compromised in a dose-dependent manner (Figure 1B; *p* = 0.0314, *p* = 0.0016, *p* = 0.0016). Given these results, we elected to use a treatment regimen of 5 µM AA for 30 min at 37 °C to induce lipid peroxidation.

### 3.2. Phospholipase Inhibition Exacerbates Lipid Peroxidation in Human Spermatozoa

**Figure 2:**
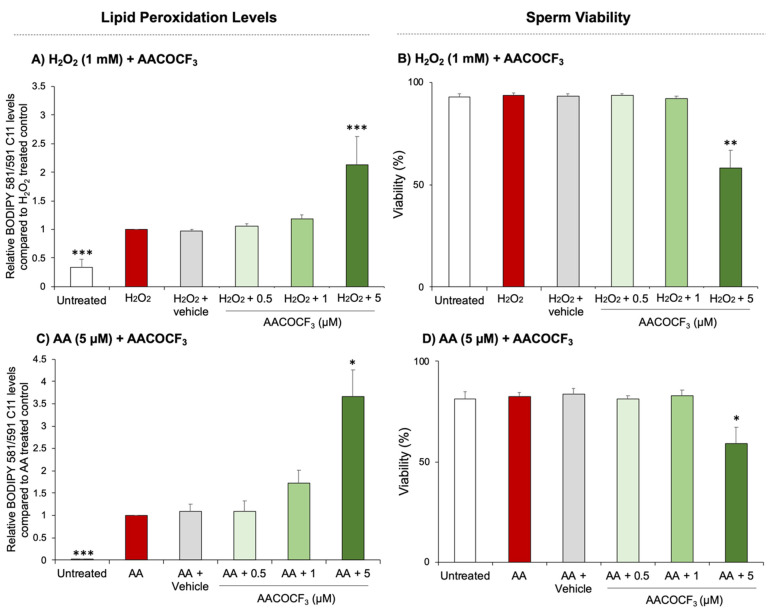
Assessment of human sperm lipid peroxidation levels following PLA inhibition. Human sperm cells were initially exposed to BODIPY 581/591 C11 (5 µM) to measure lipid peroxidation levels. Cells were then pretreated with a broad-spectrum phospholipase inhibitor AACOCF_3_ using EtOH as the vehicle control. Cells were then either treated with H_2_O_2_ (**A**): 1 mM for 1 h or arachidonic acid (**C**): 5 µM for 30 min. To ensure live cells could be distinguished (**B**,**D**), the LIVE/DEAD marker was added to each of the treatments in the final 10 min. Following this, each of the samples were washed free of the treatments and 10,000 live cells were assessed for each treatment group on the flow cytometer. JMP statistical software was implemented for statistical analysis using at least *n* = 5 biological replicates, data were normalised to the AA/H_2_O_2_ treatment and are presented as mean + SEM and *p* < 0.05 *, *p* < 0.01 ** and *p* < 0.001 ***. For lipid peroxidation levels, statistical testing compares samples with treated controls while viability levels show significant changes compared to the untreated control.

Initial mechanistic studies were designed to assess the involvement of phospholipase enzymes in the lipid peroxidation pathway of human spermatozoa. In keeping with the balance of evidence from the somatic cell literature [37,38,39], we hypothesised that phospholipase proteins, and in particular those belonging to the phospholipase A_2_ family, would be capable of liberating oxidatively damaged PUFAs from sperm membranes. To validate this hypothesis, human spermatozoa were treated with AACOCF_3_ (0.05–5 μM), a broad-spectrum inhibitor of cytosolic and calcium independent PLA_2_ [26,27]. Following pre-treatment with this inhibitor, sperm suspensions were challenged with AA to induce cellular oxidative stress and lipid peroxidation. The resulting levels of lipid peroxidation and cell viability were then objectively assessed by flow cytometry. To demonstrate the validity of the use of AA in these experiments, sperm suspensions were treated in parallel with H_2_O_2_ (as previously described [8]) and the results were compared. As demonstrated in Figure 2, both H_2_O_2_ and AA proved effective treatments for inducing significant levels of lipid peroxidation compared to the untreated controls (*p* = 0.0004 and *p* < 0.0001 respectively, Figure 2A,C). Co-treatment of sperm with H_2_O_2,_ and AACOCF_3_ yielded a significant increase in lipid peroxidation (*p* = 0.0009, Figure 2A) and was accompanied by a significant (*p* = 0.0014) loss of cell viability (Figure 2B). Similarly, the combined AA and AACOCF_3_ treatment produced a significant, dose-dependent increase in lipid peroxidation. In this instance, the highest concentration of AACOCF_3_ elicited a significant, 3.6-fold increase in lipid peroxidation above that of the AA treated control (*p* = 0.0109) (Figure 2C). Such changes were again aligned with an attenuation of sperm viability (*p* = 0.0465, Figure 2D).

### 3.3. PUFAs (AA, LA, DHA) Serve as Key Substrates for ALOX15 Driven Lipid Peroxidation in Human Spermatozoa

**Figure 3:**
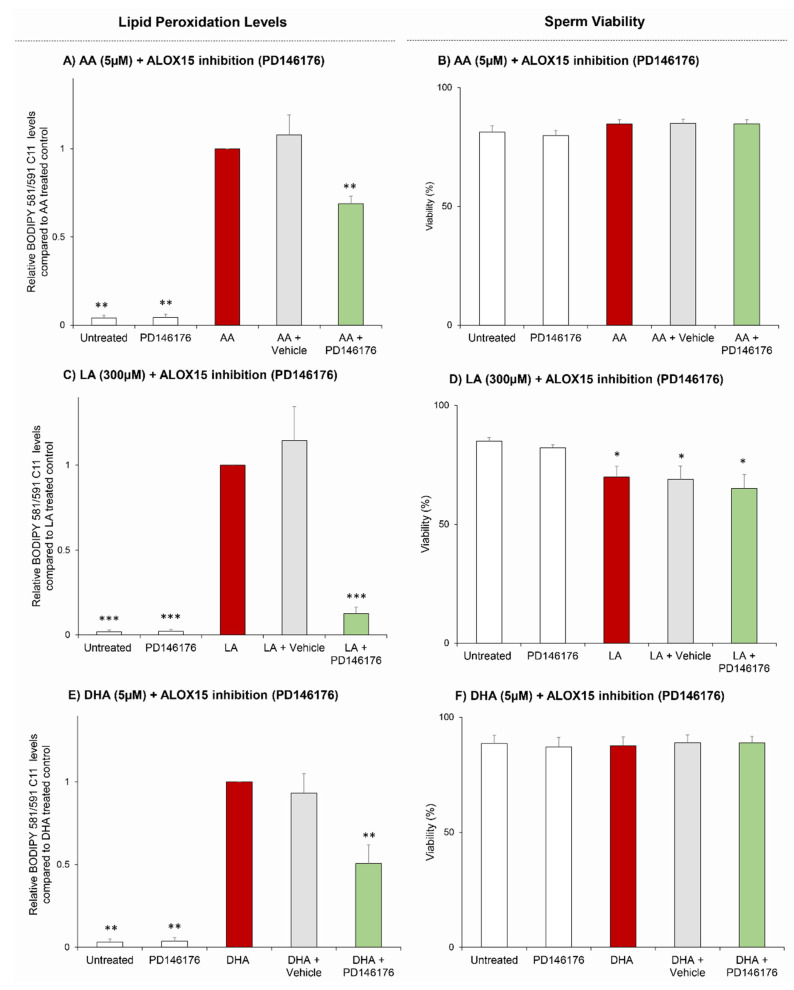
Assessment of ALOX15 PUFA substrates. Human sperm cells were incubated with BODIPY 581/591 C11 (5 µM) to provide a marker for lipid peroxidation. Samples were then pretreated with PD146176 (0.5 µM), for 30 min. Cells were then exposed for 30 min to specific PUFAs; arachidonic acid (AA)—(**A**), linoleic Acid (LA)—(**C**) and docosahexanoic acid (DHA)—(**E**). LIVE/DEAD was incubated in each of the treatments in the final 10 min to provide a viability marker (**B**,**D**,**F**). Cells were then washed free of treatments and analysed by flow cytometry. Statistical analysis was completed on at least *n* = 5 biological replicates with all data being presented as mean + SEM and with statistically significant changes being denoted by *p* < 0.05 *, *p* < 0.01 ** and *p* < 0.001 ***. Lipid peroxidation statistical analysis compared samples to the treated control (**A**,**C**,**E**), while viability assessments were compared to the untreated control.

Having established the principles by which to robustly elicit lipid peroxidation in human spermatozoa using AA and the involvement of phospholipase enzymes in this pathway, we next elected to examine the specific involvement of key PUFA substrates in evoking this response. The rationale for this study rests with independent evidence that the lipoxygenase-lipid peroxidation pathway is specifically linked to the metabolism of PUFAs, as opposed to other saturated fatty acids or neutral lipids [40,41]. Furthermore, the balance of evidence indicates that the catalytic activity of ALOX15 in somatic cells is allied to a specific subset of PUFAs, namely AA, LA and DHA, which are all present in human sperm cell membranes [27,42,43,44,45]. To assess the involvement of these PUFAs in the lipoxygenase-lipid peroxidation pathway in human spermatozoa, these cells were pretreated with the ALOX15 inhibitor PD146176 (0.5 μM) prior to the introduction of exogenous AA, LA, or DHA (Figure 3). Importantly, each of these three PUFA treatments proved effective in the induction of cellular lipid peroxidation above that of the basal levels present in untreated control samples (AA, LA, DHA: *p* = 0.0028, *p* < 0.0001, *p* = 0.0075, respectively) (Figure 3A,C,E). Furthermore, PD146176-mediated inhibition of ALOX15 significantly attenuated lipid peroxidation levels in all three treatment groups (AA, LA, DHA: *p* = 0.0028, *p* < 0.0001, *p* = 0.0075, respectively) (Figure 3A,C,E). Notably, amongst these treatment groups, only LA led to an appreciable reduction in sperm viability (Figure 3D), a response that was not reversed by prior inhibition of ALOX15 with PD146176 (Figure 3D).

To assess the specificity of the lipoxygenase-lipid peroxidation pathway responses to PUFAs, equivalent populations of spermatozoa were challenged with dose-dependent treatments of either caprylic acid (a saturated fatty acid) or docosapentanoic acid (an alternative PUFA), neither of which are known substrates for the lipoxygenase enzyme family (Supplementary Figure S1). Importantly, neither of these treatments resulted in increased levels of lipid peroxidation.

### 3.4. ALOX15 Plays a Dominant Role in Lipoxygenase-Mediated Lipid Peroxidation Pathways in Human Spermatozoa

**Figure 4:**
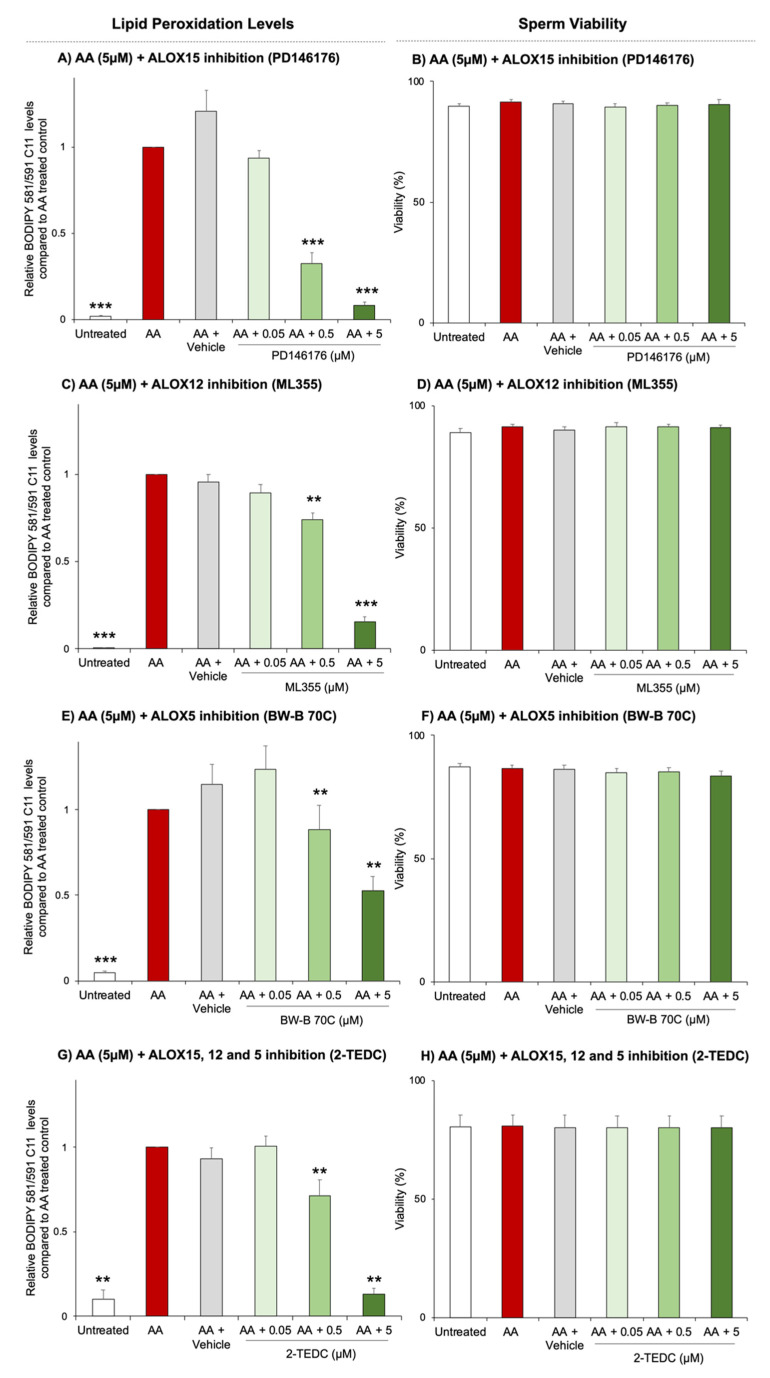
Lipid peroxidation levels in human spermatozoa following the inhibition of alternative lipoxygenase enzymes. Initially, cells were incubated with the lipid peroxidation probe BODIPY 581/591 C11 (5 µM). Lipoxygenase inhibition was then assessed using a range of lipoxygenase inhibitors (**A**): ALOX15 inhibition-PD146176, (**C**): ALOX12 inhibition—ML355, (**E**): ALOX5 inhibition—BW-B 70C and (**G**): multi lipoxygenase inhibitor—2-TEDC using DMSO as the vehicle control. Following pretreatment of each of these inhibitors for 30 min, lipid peroxidation was induced for an additional 30 min using 5 µM of arachidonic acid (AA). A viability stain of LIVE/DEAD were added to the cells in the final 10 min of this treatment. Cells were then washed free of treatments in duplicate and then 10,000 live cells were assessed by flow cytometry. Viability was assessed for each of the treatment groups (**B**,**D**,**F**,**H**). Statistical analysis was completed using at least *n* = 4 biological replicates per treatment group with data presented as mean + SEM. Statistical significance is denoted here by asterisks, *p* < 0.01 ** and *p* < 0.001 ***. For lipid peroxidation levels statistical testing compares samples with treated controls, while for viability levels show significant changes were determined compared to the untreated control.

In view of these collective findings, we sought to assess the therapeutic potential of modulating lipoxygenase activity as a means by which to protect sperm from lipid peroxidation (Figure 4). Notably, in our previous studies the inhibition of ALOX15 significantly reduces, but does not completely eliminate, lipid peroxidation levels compared to each treated control [8]. This led us to hypothesise that alternative lipoxygenase proteins may participate in the lipid peroxidation pathway in human spermatozoa. To explore the role of other members of the lipoxygenase family, the contributions of ALOX5 and ALOX12 were assessed. Of the six lipoxygenase family members, ALOX5 and ALOX12 were selected based on their tissue expression profile (both are widely expressed, whereas ALOX12B and ALOXE3 are known to be epithelial-only lipoxygenases), and the availability of specific inhibitors (there is no available ALOX15B-specific inhibitor). For the purpose of these studies, AA was used as the substrate to induce the lipid peroxidation pathway, as this PUFA can be readily processed by most members of the lipoxygenase family [9]. Targeted inhibition of ALOX12 and ALOX5 was achieved using ML355 (0.34 µM) and BW-B 70C (0.2 µM), respectively. As shown in Figure 4, both ML355 and BW-B 70C evoked significant, dose-dependent reductions in lipid peroxidation levels (Figure 4C; *p* = 0.0029 and *p* < 0.0001, Figure 4E *p* = 0.0014 and *p* = 0.0014). However, the level of inhibition achieved with ML355 and BW-B 70C was more modest than that of PD146176 (which selectively targets ALOX15) (Figure 4A,C,E). Moreover, the application of a pan-inhibitor (2-TEDC), which targets ALOX15, ALOX12 and ALOX5, albeit with varying IC_50_ values (i.e., ALOX15: 0.5 μM, ALOX12: 0.013 µM and ALOX5: 0.09 µM; values provided by Tocris Bioscience) provided additional evidence that ALOX15 may play a dominant role among the lipoxygenase enzymes examined. Thus, a significant reduction in lipid peroxidation (*p* = 0.0075) was only observed when 2-TEDC was used at concentrations greater than or equal to 0.5 μM (Figure 4G); amounts that far exceed the IC_50_ value required for selective inhibition of either ALOX5 or ALOX12; but equating to the IC_50_ value expected for ALOX15 inhibition. Importantly, none of the four inhibitors used in this study had any detrimental impact on sperm viability, which consistently remained above 80% irrespective of the treatment group (Figure 4B,D,F,H).

### 3.5. ALOX15 Protein Expression and 4HNE Protein Modifications Are Elevated in Infertile Patient Sperm Samples

**Figure 5:**
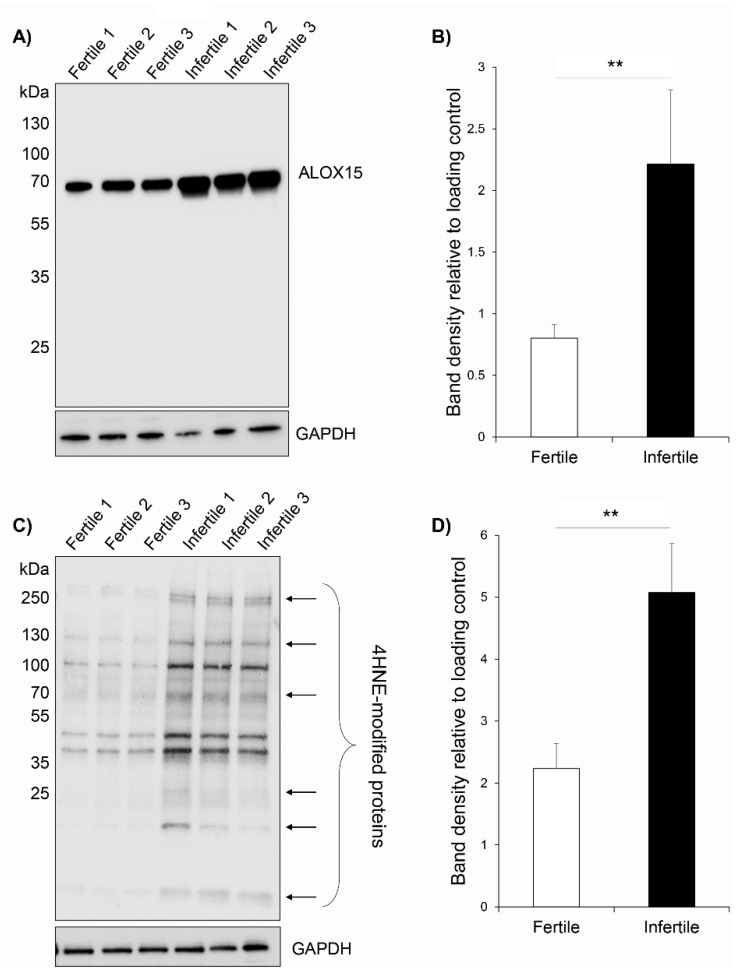
Comparison of lipid peroxidation levels and ALOX15 abundance in the spermatozoa of fertile and infertile males. Immunoblotting was used to quantify ALOX15 abundance (**A**,**B**) and 4HNE protein modifications (**C**,**D**) in sperm cell lysates obtained from infertile patients and fertile donors. Spermatozoa were isolated via Percoll or PureCeption and centrifugation protocols. Proteins were extracted and resolved by SDS-PAGE in preparation for immunoblotting with either anti-ALOX15 or anti-4HNE antibodies. Membranes were subsequently stripped and re-probed with anti-GAPDH antibodies as a loading control. Densitometry of ALOX15 and 4HNE band density was performed using Image J software. Band density was quantified to compare between infertile and infertile patient immunoblots (**B**,**D**) relative to GAPDH. Statistical analysis was performed on *n* = 3 biological and *n* = 3 technical replicates (i.e., one sample from each of three replicate donors and patients with three technical replicates performed for the immunoblotting procedure). All data are presented as mean + SEM with statistical significance denoted by ** *p* < 0.01.

Given that our data indicate that ALOX15 is the primary lipoxygenase family member involved in the catalysis of peroxidised lipids released from the human sperm membrane, we elected to examine the abundance of ALOX15 in the spermatozoa of fertile and infertile men (Figure 5). The cohort of infertile patients examined had experienced a period of infertility >2 years and possessed sperm parameters (total motility, morphology and cell number) within a ‘normal’ range according to WHO criteria (Supplementary Table S2). Any patients with tubal defects or where a female factor was identified were ruled out of this analysis. Thus, the patients used in this study received a diagnosis of ‘unexplained male infertility’. Comparison of the sperm lysates from three representative normozoospermic individuals and three representative individuals with unexplained infertility revealed a highly significant increase in ALOX15 abundance in the infertile samples (Figure 5A,B, *p* < 0.0081). To examine whether an increase in ALOX15 abundance may lead to an increase in sperm lipid peroxidation in the infertile male population, the profiles of 4HNE-modified proteins were examined via immunoblotting with anti-4HNE antibodies. This experiment revealed a significant increase in 4HNE-modified proteins in the infertile sperm lysates, displaying at least four uniquely modified protein bands compared to the fertile protein lysates (Figure 5C,D, *p* = 0.0077). These 4HNE-modified proteins ranged from ~15 to 250 kDa with a small number of constitutively modified proteins also detected in both the fertile and infertile sperm cells (~45–120 kDa; Figure 5C). This is the first experimental evidence to link ALOX15 and human sperm lipid peroxidation to a decrease in male fertility.

## 4. Discussion

This study assessed previously unexplored molecular mechanisms that underpin the lipid peroxidation pathway in human spermatozoa, with major findings implicating ALOX15 in the generation of lipid damage in sperm cells. The involvement of ALOX15 as a catalyst for lipid peroxidation was characterised via the validation of its specific substrates AA, LA and DHA and analysis of the involvement of phospholipase enzymes. Furthermore, our comparison of lipid peroxidation levels driven by other members of the lipoxygenase family, ALOX12 and ALOX5, revealed that the selective inhibition of ALOX15 appeared to be the most effective in reducing levels of lipid peroxidation in human sperm cells in vitro. Elevated ALOX15 protein abundance in men with fertility issues compared to normozoospermic donors, occurred concomitant with a significant increase in 4HNE protein modifications, an important marker of lipid peroxidation and cellular stress. Ultimately, the data generated in this study have provided an increased understanding of the lipoxygenase-lipid peroxidation pathway. Indeed, ALOX15 may be a useful marker for oxidative lipid damage and poor fertility, with the protective effects seen through pharmacological inhibition of ALOX15 highlighting the potential importance of targeting this enzyme for the prevention of oxidative stress-induced male infertility.

Phospholipase proteins are involved in the cleavage and liberation of PUFAs from phospholipids within membranes [46]. These enzymes form part of a broad family of proteins that are commonly divided among subcategories including secretory (sPLA_2_)_,_ cytosolic (cPLA_2_) and calcium independent phospholipases (iPLA_2_) [39,46]. The role of phospholipase A_2_ proteins and their role in membrane PUFA liberation and lipid peroxidation is not an extensively examined topic in the male germline, however the studies that have been completed have linked PLA_2_ proteins to fertility [47]. In particular, PLA_2_ levels have been shown to be elevated in the spermatozoa of males suffering from infertile pathologies such as azoospermia and oligospermia compared to that of their fertile counterparts [47]. Furthermore, knockout models of PLA_2_ yielding a significant depletion of the specific metabolites of ALOX15 (15-HETE, 9-HODE and 13-HODE), thus providing an important link between PLA_2_ function and lipoxygenase activity in the male germline [48].

While there are currently no established mechanistic links between PLA_2_ activity and oxidative stress in the male germline, recent studies have confirmed a direct link between the activity of the antioxidant peroxiredoxin 6 (PRDX-6) and iPLA_2_ in sperm cells [49,50]. Importantly, in vitro analysis of human sperm confirmed that targeted inhibition of iPLA_2_ and PRDX-6 resulted in increased levels of lipid peroxidation and decreased sperm function as observed through compromised acrosome reaction rates [50]. Ultimately, such results provide support for the increases in lipid peroxidation, and the loss of viability we observed following the broad-spectrum inhibition PLA_2_ proteins with AACOCF_3_ within our study. Notably, AACOCF_3_, which is marketed as a cPLA_2_ inhibitor, has also been documented to inhibit iPLA_2_ [25,26]. The increase in lipid peroxidation observed following AA/AACOCF_3_ treatment is likely to be due to the retention of peroxidised lipids left within the membrane resulting from compromised PLA_2_ activity. On the basis of these data, we propose that the inability to rapidly remove damaged lipids from the proximity of their undamaged counterparts, propagates membrane instability and may accentuate oxidative stress and cyclical lipid peroxidation. Moreover, an accumulation of lipid hydroperoxides may take place under PLA_2_ inhibitory conditions due to a decrease in detoxification [51]. This is due to detoxification enzymes present in human sperm cells, such as glutathione peroxidase 4, acting on lipid hydroperoxides that have already been released from the plasma membrane by PLA_2_ enzymes [51].

The literature from somatic cell studies indicates that ALOX15 is able to metabolise arachidonic acid (AA), linoleic acid (LA) and docosahexanoic acid (DHA) [42,43,44,45]. Our in vitro induction of lipid peroxidation using each of these PUFA substrates was extremely effective, confirming that each possessed the ability to stimulate lipid peroxidation in human sperm. Furthermore, the ability of the ALOX15 inhibitor PD146176 to dampen these responses confirmed that AA, LA and DHA can be utilised as ALOX15 substrates. Additionally, treatment of sperm cells with lipids that are not metabolised by ALOX15 (i.e., docosapentanoic acid and caprylic acid) failed to elicit any significant increase in lipid peroxidation (*p* > 0.05). Overall, these data confirm the involvement of AA, LA and DHA as specific substrates capable of fueling the lipoxygenase-lipid peroxidation pathway in human spermatozoa.

Notwithstanding these results, a modest amount of residual lipid peroxidation was consistently detected following PD146176 treatment. This observation provided an opportunity to investigate alternative members of the lipoxygenase family potentially contributing to the lipid peroxidation pathway in sperm cells. As previously reviewed [9,18,52], there are six proteins within the lipoxygenase family which include ALOX15, ALOX15B, ALOX12, ALOX5, ALOX12B and ALOXE3. These proteins are generally divided into two subgroups based on tissue expression profiles, with ALOX12B and ALOXE3 predominantly expressed in skin tissue [53,54], whereas all other members of the lipoxygenase family are documented to be more widely expressed [52]. Excluding the epithelial lipoxygenases, our analysis focused on the lipoxygenase members ALOX15, ALOX12 and ALOX5 for which specific inhibitors were commercially available. While the characterisation of these proteins within the male germline is extremely limited, ALOX5 expression has previously been linked to fertility [55]. Building on these observations, our analysis focused on the inhibition of ALOX5, ALOX15 and ALOX12 during the induction of oxidative stress in male germ cells with AA, confirming that a dose-dependent reduction in lipid peroxidation could be achieved through the inhibition of each of the three ALOX enzymes individually. However, the inhibition of ALOX5 and ALOX12, using BW-B 70C and ML355 respectively, only yielded subtle reductions in lipid peroxidation when sperm cells were exposed to the inhibitors at concentrations close to their predicted IC_50_ (BW-B 70C: 0.2 μM and ML355: 0.34 μM; in accordance with the specifications provided by each manufacturer). By comparison, ALOX15 inhibition, achieved by PD146176 treatment, revealed a dramatic reduction in lipid peroxidation at its IC_50_ concentration (0.5 μM; Tocris Bioscience) strongly supporting the notion that the ALOX15 enzyme may be amongst the dominant enzymes catalysing the sperm lipid peroxidation pathway. This conclusion was further strengthened by the response elicited by the multi lipoxygenase inhibitor 2-TEDC which had IC_50_ values of 0.09 μM (ALOX5) and 0.013 μM (ALOX12) and 0.5 μM (ALOX15), that required a supraphysiological concentration of 0.5 μM to induce a significant reduction in lipid peroxidation compared to the treated control. These data point to a likely prevailing role for ALOX15 as a master regulator within the lipid peroxidation pathway of human spermatozoa and thus highlight the merit of developing clinically safe and effective inhibitors to prevent ALOX15 action as a strategy to protect human sperm cells from oxidative damage. Notwithstanding these important findings, a caveat to this work is that the role of ALOX15B remains unknown in human sperm cells. The development of new lipoxygenase reagents, such as specific activity assays, in the future will enable the further examination of the lipoxygenase-dependent regulation of sperm membrane lipid peroxidation.

Through the assessment of patient samples, our study has revealed a significant increase in the abundance of the ALOX15 enzyme in the spermatozoa of a subset of infertile males with idiopathic infertility. Notably, elevated levels of ALOX15 detected in infertile sperm lysates were accompanied by a commensurate increase in lipid peroxidation levels in the sperm of these individuals, as measured by the abundance of 4HNE protein adducts, a common deleterious outcome downstream of lipid peroxidation. An important caveat to these findings is the use of IVF medium in the preparation of the clinical samples that may not be directly comparable in composition to the laboratory medium used to prepare fertile patient samples. While the use of IVF medium is very unlikely to impact the results described, independent studies with larger patient cohorts will be important to evaluate the infertile patient data reported within this manuscript. Notwithstanding this important caveat, to our knowledge this is the first report of ALOX15 elevation in infertile patients, which complements independent studies that have recently confirmed increases in both the ALOX15 substrate AA, and its metabolite, 15-HETE, within the seminal plasma of infertile individuals [56]. These combined data confirm the link between ALOX15 activity and oxidative lipid damage in the male germline and provide support for ALOX15 as both a potential biomarker of male infertility risk and importantly, a powerful target for preventing oxidative stress-induced male infertility.

## 5. Conclusions

Overall, this study has shed new mechanistic light on the lipoxygenase-lipid peroxidation pathway operating within human spermatozoa. Through these data, we propose that the human sperm lipid peroxidation pathway is initiated both by increases in ROS [8] and through the ALOX15-dependent oxygenation of PUFAs found in biomembranes (including AA, LA and DHA) [57]. Moreover, we have demonstrated here that the PLA_2_ family of enzymes is essential in liberating damaged PUFAs from the membrane of oxidatively stressed human spermatozoa. Without the liberation of these oxidised lipids, effective detoxification of lipid hydroperoxides by enzymes such as GPX4 is likely to be prevented. The ability of ALOX15 to additionally oxygenate free fatty acids cleaved from the membrane by PLA_2_ enzymes may also propagate lipid damage and lead to the production of cytotoxic lipid aldehydes [41,58]. This study establishes a potential link between ALOX15 and male factor infertility, demonstrated by the propensity of this enzyme to cause lipid peroxidation and an overproduction of 4HNE that features in the spermatozoa of infertile patients. These data provide a platform for the evaluation of ALOX15 abundance and activity in a larger cohort of infertile male individuals.

## Data Availability

The data presented in this manuscript and any accompanying raw data are available upon request from the corresponding author.

## Supplementary Materials

The following are available online at https://www.mdpi.com/2076-3921/10/1/43/s1, Table S1: Summary of the antibodies, fluorophores, pharmacological inhibitors and incubation media used throughout this study. Supplementary Figure S1. Lipid peroxidation levels of human spermatozoa following non-specific lipid treatment. Table S2: Sperm parameters recorded for infertile patients at time of diagnosis.
