# Supplementary material for: Mechanistic Insight into the Regulation of Lipoxygenase-Driven Lipid Peroxidation Events in Human Spermatozoa and Their Impact on Male Fertility
Source: Antioxidants (Basel). 2020 Dec 31;10(1):43. doi: 10.3390/antiox10010043 (PMC7823465; doi:10.3390/antiox10010043)
Supplement: Supplementary file 1 [file antioxidants-10-00043-s001.zip › Walters2020_supplementary tables/Table S1.docx]

**Supplementary Table S1: Summary of the antibodies, fluorophores, pharmacological inhibitors and incubation media used throughout this study**

| **Antibody, probes, inhibitors and treatments**  **(catalog number)**  **[RRID]** | **Concentration and recipe** | **Supplier** |
| --- | --- | --- |
| Anti-ALOX15 (ab80221)  [AB_1603382] | 1 µg/ml in 1% skim milk / TBST | Abcam |
| Anti-α-Tubulin (T5168)  [AB_477579] | 4.3 µg/ml in 1% BSA / TBST | Merck |
| Anti-rabbit HRP (DC03L)  [AB_10682906] | 0.13 µg/ml in 1% skim milk / TBST | Millipore |
| Anti-mouse HRP (sc-2005)  [AB_631736] | 0.4 µg/ml in 1% BSA / TBST | Santa Cruz Biotechnology |
| Anti-GAPDH (AM4300)  [AB_2536381] | 0.1 µg/ml in 1% BSA/TBST | Thermo Fisher Scientific |
| Anti-4HNE (HNE 11-S)  [AB_2629282] | 1 µg/ml in 1% skim milk / TBST | Alpha Diagnostic International |
| BODIPY 581/591 C11 (D3861) | 5 µM in NC BWW (Stock solution prepared in ethanol) | Thermo Fisher Scientific |
| LIVE/DEAD (L3224) | 1:10,000 in NC BWW (Stock solution prepared in DMSO) | Thermo Fisher Scientific |
| AACOCF_3_ (1462) | 0.05 - 5µM in NC BWW (Stock solutions of inhibitor prepared in ethanol) | Tocris |
| PD146176 (2850) | 0.05 - 5µM in NC BWW (Stock solutions of inhibitor prepared in DMSO) | Tocris |
| BW-B 70C (1304) | 0.05 - 5µM in NC BWW (Stock solutions of inhibitor prepared in DMSO) | Tocris |
| 2-TEDC (0645) | 0.05 - 5µM in NC BWW (Stock solutions of inhibitor prepared in DMSO) | Tocris |
| ML355 (18537) | 0.05 - 5µM in NC BWW (Stock solutions of inhibitor prepared in DMSO) | Cayman Chemical |
| Non-capacitating (NC) BWW | 91.5 mM NaCl, 4.6 mM KCl, 1.7 mM CaCl_2_ .2(H2 O), 1.2 mM KH_2_ PO_4_ , 1.2 mM MgSO_4_ .7H_2_O, 5.6 mM D-glucose, 0.27 mM sodium pyruvate, 44 mM sodium lactate, 5 U/ml penicillin, 5 mg/ml streptomycin and 20 mM (4-(2-Hydroxyethyl)piperazine-1-ethanesulfonic acid, N-(2- Hydroxyethyl)piperazine-N′ -(2-ethanesulfonic acid)) HEPES buffer and 1 mg/ml polyvinyl alcohol  osmolarity of 290–320 mOsm/kg | Made within the laboratory with constituents purchased from Merck |
