# Supplementary material for: Mechanistic Insight into the Regulation of Lipoxygenase-Driven Lipid Peroxidation Events in Human Spermatozoa and Their Impact on Male Fertility
Source: Antioxidants (Basel). 2020 Dec 31;10(1):43. doi: 10.3390/antiox10010043 (PMC7823465; doi:10.3390/antiox10010043)
Supplement: Supplementary file 1 [file antioxidants-10-00043-s001.zip › Walters2020_supplementary tables/Table S2.docx]

**Supplementary Table S2: Sperm parameters recorded for infertile patients at time of diagnosis**

| **Sample #** | **Patient age** | **Diagnosis** | **Sperm concentration (x10^6^/mL)** | **Volume**  **(mL)** | **Progressive motility (%)** | **Length of infertility (years)** |
| --- | --- | --- | --- | --- | --- | --- |
| 1 | 32 | Unexplained | 53 | 4.7 | 48 | 2 |
| 2 | 36 | Unexplained | 98 | 2 | 37 | 3 |
| 3 | 39 | Unexplained | 55 | 1.8 | 45 | 2 |
