# Supplementary figures and images for: Mechanistic Insight into the Regulation of Lipoxygenase-Driven Lipid Peroxidation Events in Human Spermatozoa and Their Impact on Male Fertility
Source: Antioxidants (Basel). 2020 Dec 31;10(1):43. doi: 10.3390/antiox10010043 (PMC7823465; doi:10.3390/antiox10010043)

## Lipid Peroxidation Levels

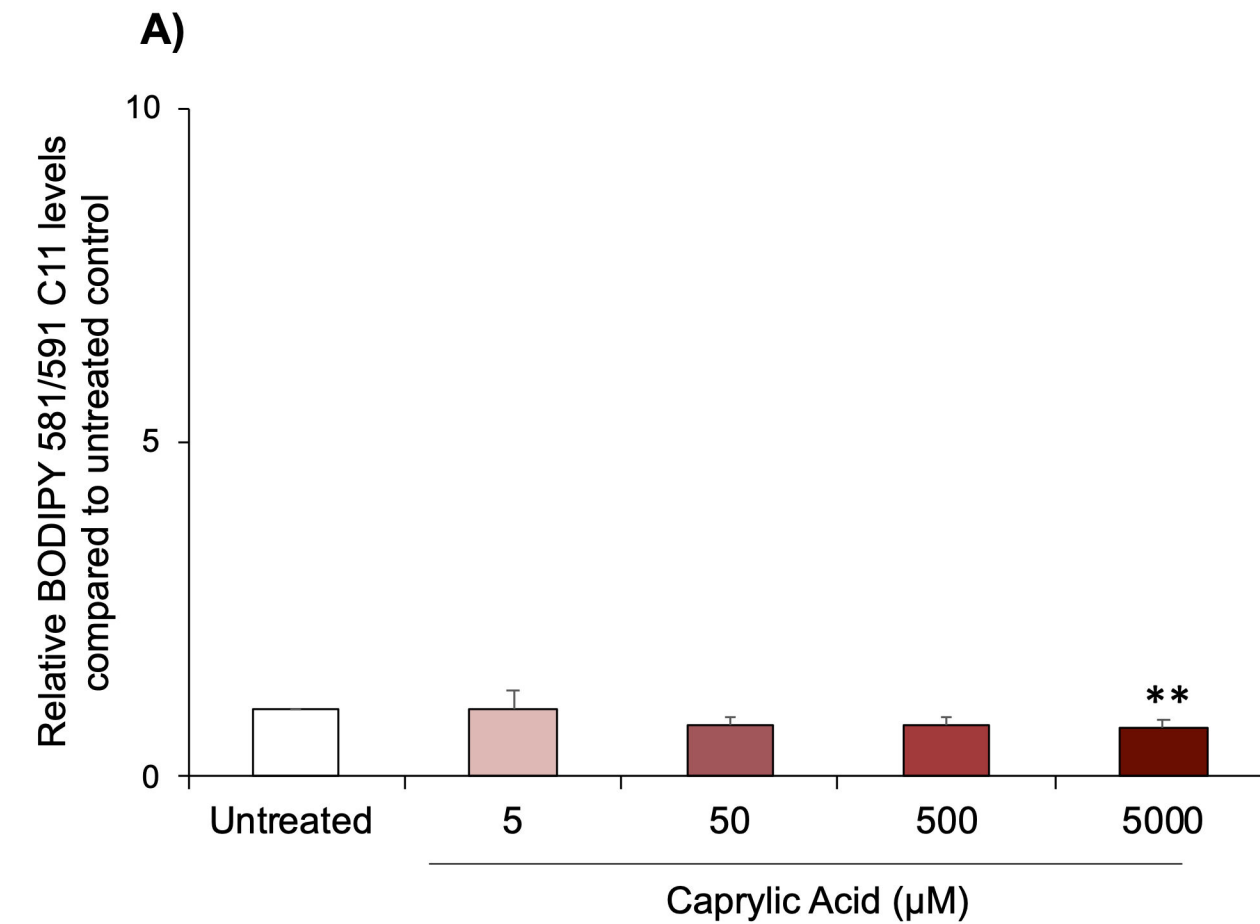

## Sperm Viability

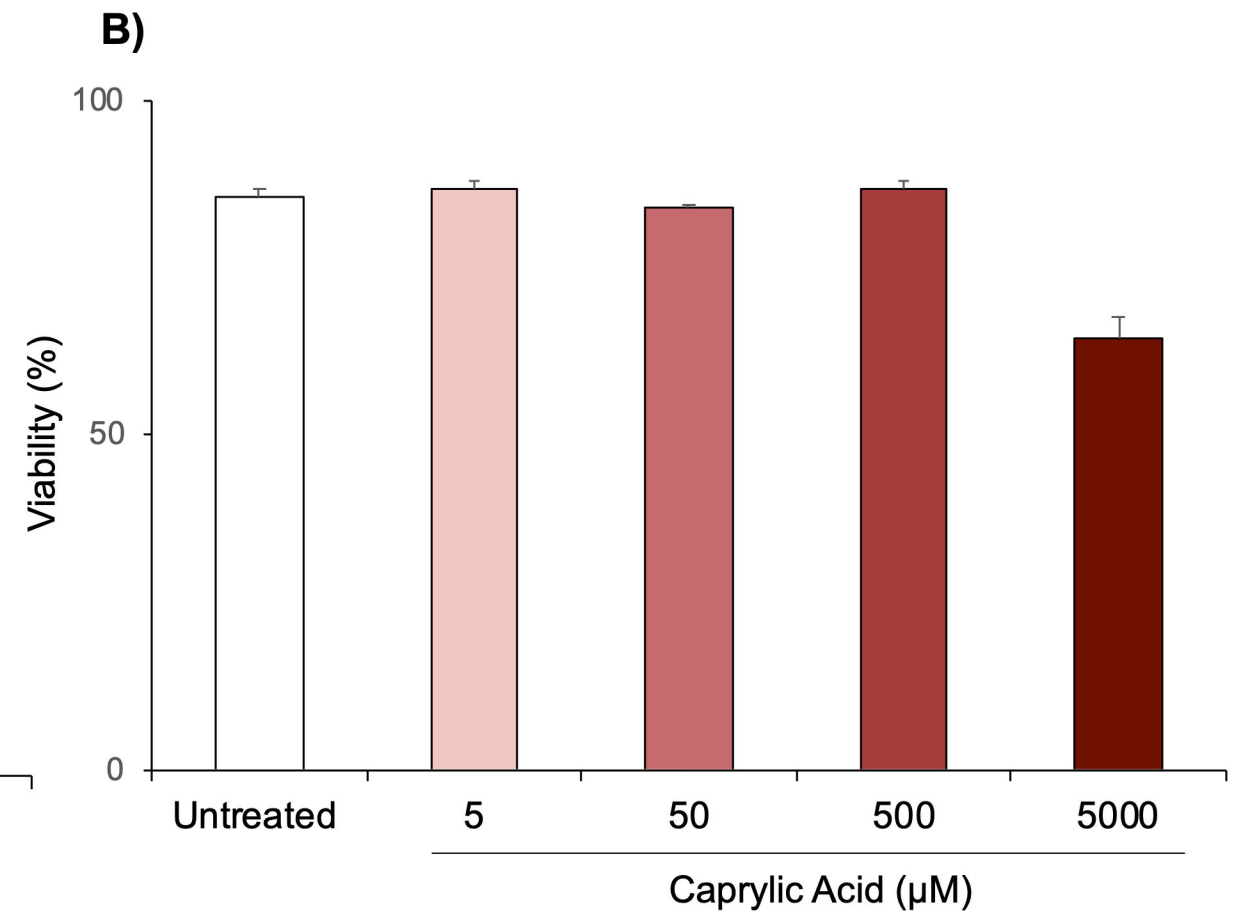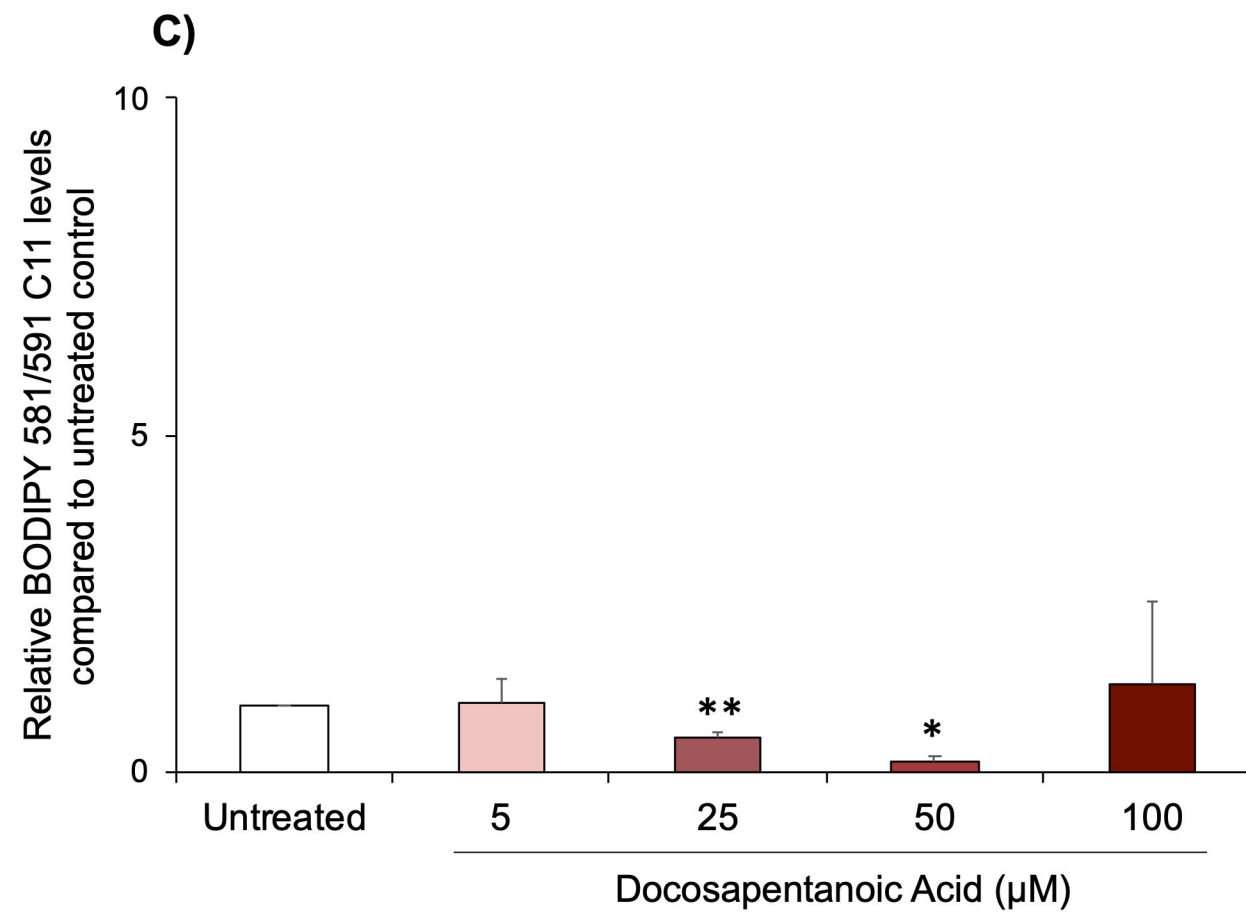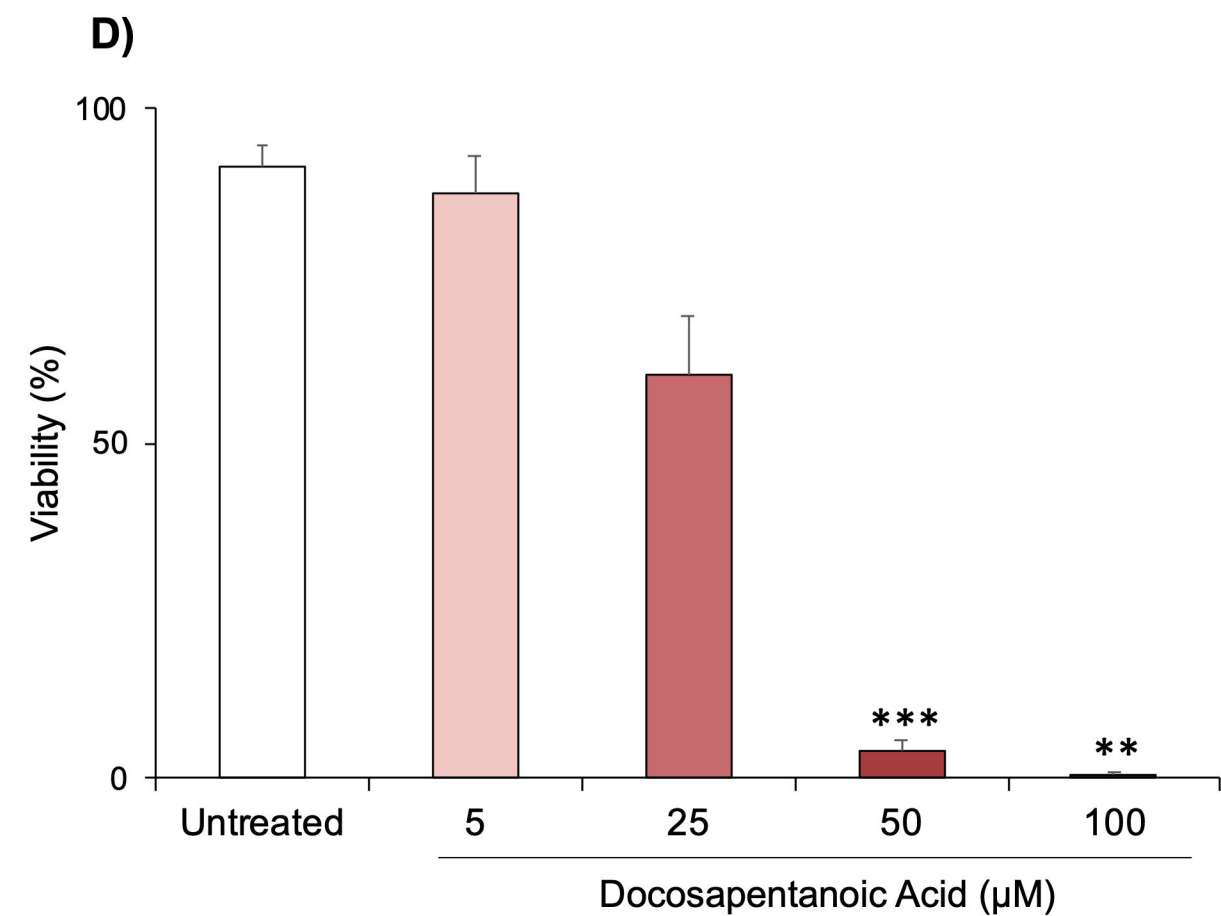

Supplement: Supplementary file 1 [file antioxidants-10-00043-s001.zip › antioxidants-1045678-supplementary figure.pdf]
